# Iron, Oxidative Stress, and Metabolic Dysfunction—Associated Steatotic Liver Disease

**DOI:** 10.3390/antiox13020208

**Published:** 2024-02-07

**Authors:** Sophie Gensluckner, Bernhard Wernly, Christian Datz, Elmar Aigner

**Affiliations:** 1 Department of Internal Medicine I, Paracelsus Medical University, Müllner Hauptstrasse 48, 5020 Salzburg, Austria; 2 Obesity Research Unit, Paracelsus Medical University, 5020 Salzburg, Austria; 3 Department of Medicine, General Hospital Oberndorf, Teaching Hospital of the Paracelsus Medical University, 5110 Oberndorf, Austria; b.wernly@kh-oberndorf.at (B.W.); c.datz@kh-oberndorf.at (C.D.)

**Keywords:** iron metabolism, oxidative stress, ferroptosis, MASLD, liver fibrosis

## Abstract

Excess free iron is a substrate for the formation of reactive oxygen species (ROS), thereby augmenting oxidative stress. Oxidative stress is a well-established cause of organ damage in the liver, the main site of iron storage. Ferroptosis, an iron-dependent mechanism of regulated cell death, has recently been gaining attention in the development of organ damage and the progression of liver disease. We therefore summarize the main mechanisms of iron metabolism, its close connection to oxidative stress and ferroptosis, and its particular relevance to disease mechanisms in metabolic-dysfunction-associated fatty liver disease and potential targets for therapy from a clinical perspective.

## 1. Introduction

Iron (Ferrum, Fe) is a metal with essential functions in cell metabolism and the physiology of the human body. These functions mostly implicate the biosynthesis of heme for proteins like hemoglobin for oxygen transport, cytochromes for redox catalysis, or iron–sulfur clusters for mitochondrial respiration. Both iron deficiency and iron overload may have detrimental consequences for health [1]. Due to this vital importance, iron metabolism is tightly regulated to cover the demand for hemoglobin biosynthesis, while, at the same time, avoiding iron toxicity [2,3]. Important particularities of iron homeostasis are its distinguished mechanisms for the systemic and cellular regulation of iron absorption, traffic, utilization, and storage on the one hand, but the lack of a distinct elimination mechanism on the other hand [4]. A tightly balanced iron homeostasis is a physiological necessity, because unbound free iron is a key contributor of toxic potential as a source of oxidative stress via the Fenton reaction and its role in ferroptosis, an iron-dependent form of regulated cell death. In metabolic-associated steatotic liver disease (MASLD), biochemical evidence of excess iron, as indicated by elevated serum ferritin concentrations, is a common finding in approximately one third of patients [5,6]. Hence, iron-induced organ damage may have a role in MASLD disease progression. This article will offer an introduction to the role of iron in oxidative stress formation and ferroptosis with regard to steatotic liver disease from a clinical perspective.

### 1.1. Iron Homeostasis

Duodenal iron uptake and release: Iron is provided by dietary intake in a heme and non-heme form. Non-heme or inorganic iron, which is derived mainly from plant-based sources, appears mostly in a ferric (Fe^3+^) form. For intestinal uptake, Fe^3+^ has to be reduced to Fe^2+^ by a ferric reductase at the apical duodenal membrane named duodenal cytochrome B (DcytB) in the presence of gastric acid. Fe^2+^ is transported to the cytoplasm via divalent metal transporter 1 (DMT1). For release on the basolateral membrane and further systemic use (mostly erythropoiesis), Fe^2+^ is exported by the only iron efflux protein ferroportin (FPN) [4,7,8]. The other dietary iron source, heme iron, is mostly found in animal food sources (meat and seafood) in hemoprotein forms like hemoglobin or myoglobin. In contrast to the absorption of non-heme iron, the uptake of heme iron remains not entirely understood. Both receptor-mediated endocytosis and membrane transport have been discussed for cellular uptake, but the detailed mechanisms of heme metabolism and transport still have to be elucidated [9].

Extracellular transport: After efflux through FPN, Fe^2+^ is oxidized to Fe^3+^ by the ferroxidases hephaestin or ceruloplasmin and loaded to transferrin (Tf). Tf is the major iron carrier of extracellular space. In the circulation, up to two Fe^3+^ atoms are bound by Tf for transport to the body’s tissues. In healthy conditions, transferrin saturation with Fe^3+^ is about one third of its total capacity, leaving a substantial buffer [4,7,8]. The amount of extracellular Tf-bound iron accounts for approximately 0.1% (3 mg) of the total body iron (3–5 g). The fraction of dietary absorbed iron contributes approximately 1–2 mg per day, compensating for non-specific daily loss through bleeding or cell desquamation; therefore, iron supply for erythropoiesis (needs of 25–30 mg/d) via circulating Tf is mostly generated by iron recycled from senescent red blood cells via heme catabolism in macrophages [4].

Cellular iron uptake, intracellular use and storage: At its target site, iron-loaded Tf binds to ubiquitous transferrin receptor 1 (TfR1). The expression of TfR1 is high in erythrocyte precursor cells in the bone marrow due to permanent iron demand in erythropoesis. The variant Tf receptor 2 (TfR2) is found on hepatocytes and erythoid precursor cells, where it has a role in iron regulation and may serve as a default iron uptake mechanism of low physiological importance. The complex of Tf and TfR1 is internalized by endocytosis. Within the endosome, Fe^3+^ is reduced to Fe^2+^ by metalloreductase STEAP3 (six-transmembrane epithelial antigen of prostate 3). The reduced Fe^2+^ is exported from the endosome to the cytosol via DMT1 and may be further utilized or stored while TfR1 is released back to cell surface [10].

With the endosomal release of Fe^2+^ to the cytosol, iron enters the so-called labile iron pool and is used directly for intracellular mechanisms like mitochondrial protein biosynthesis by incorporation into heme or an iron–sulfate cluster or is stored within the cell as ferritin. Ferritin is a protein complex of 24 subunits of heavy- and light-chains. The heavy chains have ferroxidase activity and provide the oxidation of Fe^2+^ to its non-toxic Fe^3+^ forms for storage [4,7,8,11].

Intracellular iron regulation: Intracellular iron regulation is mostly facilitated by iron regulatory proteins (IRP) and the hypoxia-inducible factor (HIF) system, which regulate gene translation and transcription, while the most important systemic regulatory mechanism is the so-called hepcidin–ferroportin axis. Hepcidin is a protein derived from the liver, which acts as negative feedback on iron increases in the circulation by the inhibition of iron release from the enterocytes and macrophages. It binds to FPN and causes the internalization and degradation of this only export protein, therefore blocking iron efflux from the cells. Hepcidin expression increases in states of iron overload to prevent further iron release, as well as in inflammation to reduce bioavailability for iron-dependent pathogens. This inflammatory mechanism of hepcidin increase is a key mechanism of anemia in inflammation/chronic disease. The expression of hepcidin is decreased in states of high iron demand like iron deficiency, anemia, or hypoxia [12,13].

### 1.2. Iron, Oxidative Stress, and Ferroptosis

Oxidative stress: Redox reactions (oxidation and reduction) are fundamental mechanisms in cell homeostasis. Reactive oxygen species (ROS) are a byproduct of multiple redox reactions. The term ROS refers to a diversity of molecules, which include non-radicals (hydrogen peroxide H_2_O_2_ or ozone O_3_, e.g.) as well as free radicals like superoxide anion radical (O_2_^•^^−^) and hydroxyl radical (^•^OH). While oxidative stress generally describes common and essential reversible oxidative modifications (redox regulation and signaling, e.g.), it may also refer to an imbalance of oxidants and antioxidants and the possibility of molecular and cellular damage by an accumulating toxic burden caused by excessive oxidative reagents [14].

Iron metabolism and oxidative stress: Iron plays a key role in redox signaling and oxidative stress by catalyzing the Fenton reaction. The Fenton reaction generates a hydroxyl anion (^•^OH) and hydroxide ion (OH^−^) from hydrogen peroxide (H_2_O_2_) via the oxidation of Fe^2+^ to Fe^3+^. In order to avoid toxicity via increasing ROS through the Fenton reaction, iron is kept safely stored as ferritin in the intracellular space and bound to transferrin in the extracellular space [15]. An accumulation of excess iron within the cell results in an increase in the labile iron pool of redox-active Fe^2+^, which may further contribute to the formation of reactive free radicals [16]. In the extracellular space, an accumulation of excess iron leads to an increased fraction of non-transferrin bound iron (NTBI). NTBI can enter the cell via a ZIP14 transporter and act as a source of ROS [17]. Especially in hepatocytes and pancreatic acinar cells, ZIP14 (SLC39A14) was identified as the main carrier for NTBI intake during iron overload [18].

The generation of ROS was identified as part of the pathogenesis in common diseases like metabolic-associated fatty liver disease or type 2 diabetes, where excess free fatty acids lead to changes in mitochondrial function, ER stress, or NADPH oxidases with the potential accumulation of ROS and further disturbances in the lipid metabolism and insulin signaling [19]. Harmful mechanisms induce lipid and amino acid peroxidation, leading to further lipid, protein, or DNA damage and, following overwhelmed repair mechanisms, even to regulated cell death (apoptosis) [16,20]. Thereby, an excessive generation of ROS may have detrimental effects on many organs, including the liver, heart, kidney, pancreas, or nervous system [20].

Oxidative stress and ferroptosis: Another newly recognized, non-apoptotic, iron-dependent form of regulated cell death was described in 2012 and termed ferroptosis [21]. It refers to an iron-dependent accumulation of toxic lipid ROS via lipid peroxidation and a loss of the lipid repair enzyme glutathione peroxidase 4 (GPX4) due to glutathione shortage as a result of impaired cystine import [22,23].

**Figure 1:**
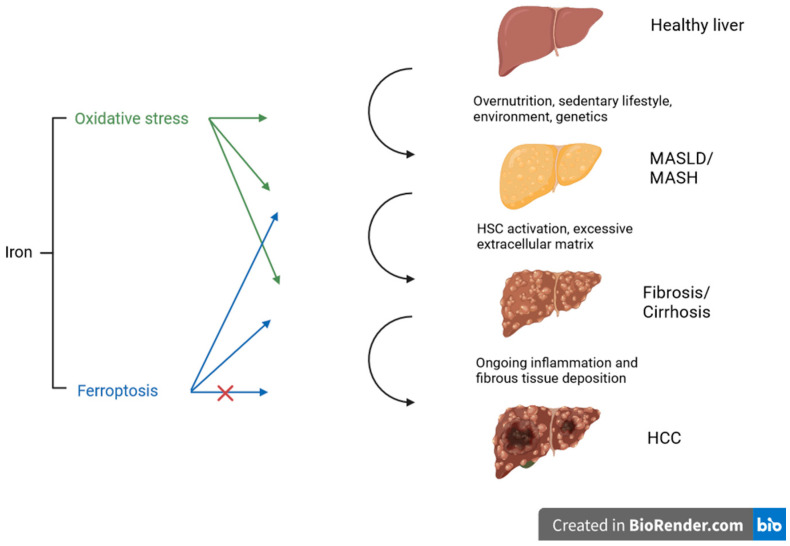
Schematic overview of different stages of liver disease influenced by oxidative stress and ferroptosis. Both mechanisms promote steatosis, inflammation, and fibrosis, indicated by the arrows. In tumor development, ferroptosis may induce cancer cell death and may therefore serve as an anticancer strategy (the potential inhibitory influence is indicated with the red cross), representing a potential target for therapy. Created with BioRender.com.

Under normal circumstances, cystine is transported into the cell via the antiporter system Xc^−^ and used for the synthesis of glutathione (GSH). GSH is necessary for the function and activity of glutathione peroxidase 4 (GPX4) and represents an important antioxidant mechanism in the cell. GPX4 reduces and neutralizes lipid peroxides, which are the product of the lipid peroxidation of the polyunsatured fatty acids (PUFA) of membrane lipids. The accumulation of lipid peroxides and further iron-dependent generation of ROS are the key characteristics of ferroptosis, though the underlying initiation of lipid peroxide formation is unclear. There are different mechanisms or categories leading to the accumulation of lipid peroxides, causing the degradation, inactivation, or inhibition of GPX4 or the inhibition of cysteine import. Excess lipid peroxides represent an abundant source for Fenton-like reactions with ferrous or ferric iron, creating toxic lipid ROS and further leading to ferroptosis [24,25]. In recent research, the mechanism of ferroptosis was linked to diabetes complications [26], cardiovascular disease [27], organ fibrosis (liver, lung, heart, and kidney, e.g.) [28,29], and also liver disease [30]. Figure 1 illustrates the impact of oxidative stress and ferroptosis on the progression of steatotic liver disease at different stages. Several mechanisms will be discussed in more detail in the following sections.

## 2. Oxidative Stress and Metabolic-Dysfunction-Associated Steatotic Liver Disease

MASLD and MASH: Steatotic liver disease (SLD) describes a disease spectrum from simple liver steatosis (macrovesicular steatosis in >5% hepatocytes) to steatohepatitis (steatosis in combination with inflammation and hepatocellular ballooning). Further disease progression may lead to liver fibrosis, cirrhosis, and hepatocellular carcinoma. The primary causes of fatty liver disease are overnutrition and a sedentary lifestyle, while additional environmental and genetic factors may contribute to disease progression [31]. Since there is often an underlying metabolic dysfunction associated with it and distinction from alcohol-related liver disease is difficult, a change in nomenclature to metabolic-associated fatty liver disease (MAFLD) has been proposed [32]. For the reasons of a more inclusive and broadly accepted definition and less stigmatizing language, the terminology has recently been refined to metabolic-dysfunction-associated steatotic liver disease (MASLD), metabolic-dysfunction-associated steatohepatitis (MASH), and MetALD for those with MASLD in combination with increased alcohol intake [33].

MASLD and oxidative stress: In the development of MASLD and MASH, oxidative stress is one contributing factor to insulin resistance, inflammation, lipotoxicity, and stellate cell activation [34,35]. In insulin resistance, which represents the key mechanism in MASLD, excess free fatty acids cause an upregulation of mitochondrial citric acid cycle activity, leading to an increased generation of ROS and lipid peroxidation, causing hepatocellular damage [36]. In MASLD, different markers of oxidative stress have been identified in higher levels, as well as antioxidant molecules to counteract the burden of oxidative stress [37]. Oxidative stress is also an established mechanism in the progression from MASLD to MASH [38]. Indeed, an NAS score of ≥4 was associated with higher levels of lipid peroxidation products in a study of 152 patients with steatotic liver disease, suggesting a clinically relevant contribution of oxidative damage to disease progression [39]. Due to iron’s pro-oxidizing abilities, iron and iron overload are considered to be important contributors to the production of ROS and development of MASLD and MASH [40,41]. A greater iron accumulation and aggravated steatohepatitis were found in mice in which the function of Nrf2 (nuclear factor erythroid 2-related factor 2), which usually serves antioxidant response mechanisms, was knocked out [42]. Similarly, another model of dietary iron-fed mice showed increased levels of lipid peroxidation derivates and an increased expression of antioxidant genes as a sign for increased hepatic oxidative stress, as well as a significantly more severe MASH histology [43]. Nevertheless, to our knowledge, no clinical study has linked the parameters of iron metabolism, oxidative stress, and MASLD and MASH in vivo so far.

MASLD and ferroptosis: Because ferroptosis is based on iron overload and lipid peroxidation, it is intriguing to study its causative role in the development of MASLD and MASH. Elevated serum ferritin levels are found in approximately one third of subjects with fatty liver disease [44]. The finding of elevated serum ferritin levels and iron excess in liver biopsies accompanied with metabolic diseases like MASLD, type 2 diabetes, or obesity was termed dysmetabolic iron overload syndrome (DIOS) in 1997 [45], while recently, a consensus statement harmonized and standardized the definition as metabolic hyperferritinemia (MHF) [46]. Yet, the complex underlying mechanisms of MHF have not been entirely elucidated. However, there is growing evidence of MHF correlating with increased hepatic iron deposition [6] and disease severity [5,47], while increased inflammatory activity may further increase serum ferritin concentrations [48]. In MASLD, different cell death mechanisms like necroptosis, pyroptosis, and ferroptosis contribute to disease progression [49]. Tsurusaki et al. showed that ferroptosis precedes other types of cell death, suggesting a potential role of ferroptosis as a trigger in the development of MASH. Furthermore, they demonstrated that two inhibitors of ferroptosis (Trolox, a vitamin E analog, and deferiprone, an iron chelator) suppressed inflammation and cell death, encouraging the potential therapeutic relevance of ferroptosis inhibition [50]. In line with these findings, ferroptosis and iron accumulation were found in diet-induced murine MASH [51]. Another mouse model demonstrated that ferroptosis increased the severity of MASH, while the inhibition of ferroptosis decreased disease severity. Likewise, the use of an iron chelator (desferoxamine) inhibited the progression of MASH [52]. More recently, an analysis of a small sample of human liver tissues also indicated that ferroptosis was activated in MASLD. The study identified different GPX4 isoforms with opposing effects on ferroptosis in hepatocytes, indicating the complex regulation of ferroptosis [53]. Both ferroptosis inducers and inhibitors provide promising therapeutic opportunities along the spectrum of MASLD to hepatocellular carcinoma [54]. Nevertheless, the underlying mechanisms in ferroptosis and their iron-related effects on MASLD progression still have to be studied in detail [55]. A recent investigation of Zhang et al. described the protective effects of metformin in MAFLD related to type 2 diabetes mellitus and linked these effects to the negative regulation of ferroptosis caused by the modulation of a GPX4 pathway [56]. Still, the distinct role of iron metabolism in ferroptosis has to be elucidated, although many regulatory proteins involved in iron homeostasis, like ferritin heavy and light chains, heme oxygenase 1 (HO-1), and transferrin, have been identified to modulate ferroptosis [57]. Recent research has also focused on the identification of ferroptosis-related genes to reveal a better understanding of associated disease mechanisms and for the development of potential diagnostic and prognostic models. For MASLD, ferroptosis-related genes which correlated with diagnosis and prognosis, as well as with immune cell infiltration, were described in two different MASLD subtypes [58].

## 3. Oxidative Stress and Liver Fibrosis

Liver fibrosis: Fibrosis is the result of the excessive production of extracellular matrix, independent of the underlying cause. Metabolic liver diseases (alcoholic and non-alcoholic) may initiate this process, as well as other toxic, viral, autoimmune, or genetic etiologies. Organ damage leads to the release of damage-associated patterns (DAMPs) from apoptosis, which initiate the key mechanistic step of fibrogenesis, i.e., the activation of quiescent hepatic stellate cells (HSC) to pro-fibrogenic, collagen-producing myofibroblasts [59].

Liver fibrosis and oxidative stress: ROS from oxidative stress have been identified as one underlying pathway in liver fibrosis. In the pathogenesis of liver fibrosis, ROS may initiate the TGF-β (transforming growth factor β) signaling pathway, with further activation of SMAD (small mother against decapentaplegic) proteins and the generation of extracellular matrix, which is considered to be one of the most important pathways in fibrogenesis [60]. Within this pathway, ROS and various isoforms of NOX (NADPH oxidase) have been linked to hepatic stellate cell activation [61]. Furthermore, the generation of ROS is usually blocked by NF-κB, though this represents only one among other important functions of NF-κB in the regulation of hepatic stellate cells and liver fibrosis [62]. A recent model of human and mouse HSC even showed that iron accumulation induces fibrosis in an ROS-dependent manner [63]. Several additional mechanisms of iron accumulation as a contributing factor in fibrogenesis have been described, like cross-connection with the TGF-β signaling pathway, iron-related protein–receptor complexes, and further HSC activation or iron-induced inflammation [64].

Liver fibrosis and ferroptosis: Ferroptosis is also recognized as an underlying mechanism for fibrosis [65], since the anti-fibrotic effect of magnesium isoglycyrrhizinate was linked to the ferroptosis of HSC via an HO-1 dependent mechanism. Many other regulators of ferroptosis in HSC have been identified so far [66,67]. In transferrin knockout mice fed with a high iron diet, liver damage caused by iron-induced ferroptosis was increased, while liver fibrosis was reduced when ferroptosis was blocked. Additionally, liver biopsy samples of cirrhotic patients with low levels of transferrin had higher iron deposits and higher levels of lipid peroxidation [68]. Another animal model found that fibroblast growth factor 21 (FGF21), which is considered to be an important hormone in stress response, was induced by iron overload and that the activation of FGF21 inhibited ferroptosis in hepatocytes. Furthermore, the overexpression of FGF21 attenuated liver fibrosis induced by iron overload, suggesting a suppression of ferroptosis via FGF21 and heme oxygenase 1 [69].

The effect of various compounds on ferroptosis in hepatic stellate cells has been investigated. For example, sorafenib was identified as an inducer of ferroptosis and ferroptotic events were involved in the inhibition of HSC activation via sorafenib in vitro. Additionally, sorafenib-induced ferroptosis attenuated liver fibrosis in a mouse model [70]. Also, derivatives from a Chinese plant (Artemisin) triggered ferroptosis in activated HSC in vitro and in fibrotic livers in mice [71], while another study showed that an Artemisin derivative regulated ferroptosis in HSC via a p53-dependent mechanism [72]. Berberine, another phytopharmaceutical compound, showed anti-fibrotic properties after mediating HSC ferroptosis [73], as well as curcumol, the main active moiety of curcuma [74]. All these studies suggest that ferroptosis serves as a clinically relevant mechanism in the development of liver fibrosis. Although most findings are based on in vitro studies and experimental design, they may help in designing clinical studies targeting ferroptosis for developing potential therapies in liver fibrosis.

## 4. Oxidative Stress and Hepatocellular Carcinoma (HCC)

Hepatocellular carcinoma: The development of HCC is mostly associated with underlying chronic liver disease with an ongoing inflammatory response and fibrous tissue deposition, and the majority is found in patients with liver cirrhosis of any cause [75]. Owing to the growing prevalence of obesity and diabetes worldwide, an increasing number of HCC is found in patients with MASLD and MASH, and up to one third of MASLD-related liver cancer is found in a pre-cirrhotic stage. Patients with MASLD-associated HCC tend to be older in age, have a higher BMI, present with metabolic comorbidities like diabetes, arterial hypertension, or dyslipidemia more often, and are more likely to present with uninodular lesions with a larger tumor diameter [76,77]. Steatotic liver disease is associated with an increased risk for the development of HCC even without cirrhosis, with risk factors of obesity, type 2 diabetes, and genetic variants (PNPLA3, MBOAT7, and TM6SF2, e.g.) [78].

HCC and oxidative stress: Hepatocarcinogenesis is a complex multistep mechanism of the initiation, promotion, and progression of cellular, molecular, and genetic alterations in a certain tumor microenvironment, with oxidative stress as an established trigger and driver [79]. In obesity-related HCC, different ROS-mediated pathways have been associated with carcinogenesis. Nrf2, which usually protects against oxidative stress, as mentioned above, might suppress or promote tumor growth, depending on the state of activation. Under normal conditions, Nrf2 is bound to KEAP1 (Kelch-like ECH-associated protein 1) in the cytoplasm. During oxidative stress, the accumulation of ROS leads to the degradation of KEAP1 and Nrf2, allowing the latter to translocate to the nucleus to activate the transcription of several antioxidant protective genes [80,81]. On the other hand, these antioxidative mechanisms may also serve to protect tumor cells and therefore provide oncogenic abilities. Additionally, the activation of Nrf2 in cancer cells leads to a redistribution of glucose and further cell growth [82,83]. Also, the subsequent activation of an Nrf2/PINK1 (PTEN-induced kinase 1) pathway following a sudden increase in ROS after incomplete radiofrequency thermal ablation (RFA) resulted in a pro-survival effect in tumor cells in vitro, explaining a potential mechanism of HCC resistance after RFA [84].

Similarly, NF-κB has pro- and antioxidant properties and opposite functions in carcinogenesis, depending on its timing and activation. A signaling pathway via ROS and NF-κB, activated by mitochondrial ribosome CRIF1 (CR6-interacting factor), was associated with tumor growth and metastasis in HCC [85]. The overexpression of another protein for mitochondrial transcription, TFB2M (Mitochondrial transcription factor B2), activated ROS-Akt-NF-κB signaling and was also linked to HCC cell growth and metastasis [86]. In obesity-related HCC, the initiation of tumor suppressor gene p53 and HIF has also been linked to tumor development, as well as the BCL2 protein family, with pro- and antiapoptotic functions or the ROS-mediated inactivation of protein tyrosine phosphatase, which also plays a key role in insulin resistance [81]. Interestingly, iron also seems to act as a directly hepatocarcinogenic agent via ROS formation and oxidative stress as well, even in the absence of fibrosis or cirrhosis [87]. Besides this direct effect, several studies have identified iron overload as an additional risk factor in the development of HCC in other underlying liver disease etiologies as well [88]. The association of iron overload and tumor development has especially been studied in genetic diseases with primary iron overload like hereditary hemochromatosis. In the vast majority of patients, iron overload arises from a loss of function mutation in the HFE gene. This results in insufficient production of hepcidin, the hormone that inhibits cellular iron release through its interaction with the iron exporter ferroportin, as mentioned above. The loss of hepcidin function results in excess iron release and uptake, leading to systemic iron overload and potential organ damage. Interestingly, not every individual with biochemical signs of iron overload develops organ damage, indicating the importance of antioxidant defense mechanisms and the influence of other metabolic co-factors in disease progression [89]. Hemochromatosis is associated with an increased risk for HCC, not just due to liver disease progression to severe fibrosis and cirrhosis. An increased risk was also found in patients without fibrosis, indicated by low APRI values (AST to platelet ratio index) but constantly elevated serum ferritin levels [90].

HCC and ferroptosis: The induction of cell death is a common anticancer strategy. Therefore, ferroptosis, as a special form of metabolically induced cell death, is increasingly recognized as a tumor suppression mechanism [91]. Despite the abovementioned role of ferroptosis in the development of metabolic liver disease, the mechanism of this unique kind of regulated cell death also seems to be an attractive therapeutic target, especially in HCC [92]. Proteins and enzymes that have been involved in the regulation of ferroptosis in HCC include Nrf2, S1R (Sigma-1 receptor), HSPB1 (heat shock protein beta-1), p53, and YAP/TAZ (Yes-Associated Protein/Transcriptional Co-Activator With PDZ-Binding Motif), e.g., mostly influencing resistance to or the negative regulation of ferroptosis [93]. The identification of inducers or inhibitors of ferroptosis in HCC is still limited to basic research, and the further translation of these complex associations to clinical scenarios or even to clinical use is needed [94]. For example, sorafenib is an established inducer of ferroptosis with anti-cancer properties, but due to heterogenous tumor biology and resistance mechanisms, efficacy in HCC treatment is variable [95,96]. Recent investigations have shown that metformin also induced ferroptosis in HCC and decreased sorafenib resistance via an ATF4/STAT3 pathway in a cell culture and mouse model [97]. Furthermore, a combination of metformin and sorafenib increased tumor suppression through ferroptosis via an Nrf2-mediated pathway in HCC [98]. Orlistat, an inhibitor of fatty acid synthase, also showed anticancer effects in combination with sorafenib via ferroptosis [99]. A very interesting treatment approach for HCC called TAFE (transarterial ferro-embolization therapy) was recently described by Wang et al., combining ferroptosis-inducing agents with transarterial chemoembolization in a mouse model. The investigators prepared a Pickering emulsion of Lipodiol, calcium carbonate nanoparticles, hemin, and lipoxygenase (LHCa-LPE). This agent could effectively induce ferroptosis and tumor suppression in the cell model. After transarterial administration, LHCa-LPE also showed better tumor proliferation control and a more effective tumor inhibitory effect, resulting in a smaller tumor size and better median survival in a mouse model. This treatment shows promising results, combining the embolic effect of transarterial tumor treatment with local ferroptosis-inducing properties [100].

## 5. Conclusions

In conclusion, iron and oxidative stress have essential functions in cell biology and physiology, but carry a harmful potential, especially in states of overload. There is a tight connection and interplay between iron and oxidative stress and their influences on metabolic liver disease and liver fibrosis. Finally, a recently discovered form of iron-dependent regulated cell death called ferroptosis provides further insights into the pathogenesis of liver diseases and their progression, and may represent a target in the development of antisteatotic, antifibrotic, and anticancer therapies.

## Data Availability

Not applicable.
